# The luminal Ca^2+^ chelator, TPEN, inhibits NAADP-induced Ca^2+^ release

**DOI:** 10.1016/j.ceca.2012.09.001

**Published:** 2012-12

**Authors:** Anthony J. Morgan, John Parrington, Antony Galione

**Affiliations:** Department of Pharmacology, University of Oxford, Mansfield Road, Oxford OX1 3QT, UK

**Keywords:** NAADP, Luminal Ca^2+^, TPC, Acridine orange, IP_3_, Sea urchin, Ionomycin, Nigericin, TPEN, Leak, NH_4_Cl

## Abstract

The regulation of Ca^2+^ release by luminal Ca^2+^ has been well studied for the ryanodine and IP_3_ receptors but has been less clear for the NAADP-regulated channel. In view of conflicting reports, we have re-examined the issue by manipulating luminal Ca^2+^ with the membrane-permeant, low affinity Ca^2+^ buffer, TPEN, and monitoring NAADP-induced Ca^2+^ release in sea urchin egg homogenate. NAADP-induced Ca^2+^ release was almost entirely blocked by TPEN (IC_50_ 17–25 μM) which suppressed the maximal extent of Ca^2+^ release without altering NAADP sensitivity. In contrast, Ca^2+^ release via IP_3_ receptors was 3- to 30-fold less sensitive to TPEN whereas that evoked by ionomycin was essentially unaffected. The effect of TPEN on NAADP-induced Ca^2+^ release was not due to an increase in the luminal pH or chelation of trace metals since it could not be mimicked by NH_4_Cl or phenanthroline. The fact that TPEN had no effect upon ionophore-induced Ca^2+^ release also argued against a substantial reduction in the driving force for Ca^2+^ efflux. We propose that, in the sea urchin egg, luminal Ca^2+^ is important for gating native NAADP-regulated two-pore channels.

## 1 Introduction

Ca^2+^ release channels on intracellular stores are not only subject to regulation by second messengers but also by additional factors that include accessory proteins, pH and phosphorylation [1–5]. Of primary importance is the exquisite regulation of the channels by Ca^2+^ itself, a feedback that is essential for generating the hierarchy of Ca^2+^ signals such as local release events, Ca^2+^ oscillations or Ca^2+^ waves [6]. This feedback is multifaceted and is not restricted to one site on a given channel: Ca^2+^ can stimulate or inhibit channel activity since there can be multiple Ca^2+^-binding sites on the channel complex, some on the cytosolic face, others on the luminal face.

Cytosolic Ca^2+^ is well accepted to stimulate or inhibit inositol 1,4,5-trisphosphate (IP_3_) receptors (IP_3_Rs) and ryanodine receptors (RyR) as it follows a bell-shaped concentration response curve [5,6]. By contrast, the NAADP (nicotinic acid adenine dinucleotide phosphate) receptor has hitherto been reported to be insensitive to cytosolic Ca^2+^ (or surrogate ions) [1,7–9] and therefore local, “trigger” Ca^2+^ released by NAADP is necessarily amplified by proximal IP_3_Rs or RyRs which are Ca^2+^-sensitive [10,11].

Whether these Ca^2+^-release channel families are regulated by Ca^2+^ within the lumen of the stores themselves is more controversial [12]. For IP_3_Rs and RyRs, higher luminal Ca^2+^ concentrations promote channel opening, possibly via intermediate luminal Ca^2+^-binding proteins [5,6,12,13]. However, NAADP-regulated channels were initially reported to be insensitive to luminal Ca^2+^ [9]. More recently, mammalian members of the TPC (two-pore channel) family – the newly discovered target of NAADP [14–16] – exhibited sensitivity to luminal Ca^2+^ whereby increasing luminal Ca^2+^ concentrations enhanced channel activity in lipid bilayers [17,18]. Although plant TPC has not yet been shown to be modulated by NAADP, the channel is also influenced by luminal Ca^2+^, albeit in an inhibitory manner [19].

In view of the potential confusion surrounding these disparate results, we have re-examined the role of luminal Ca^2+^ in regulating NAADP responses in sea urchin egg, a system in which TPCs are channels regulated by NAADP [20,21] possibly via smaller accessory proteins that are the NAADP-binding moieties [22,23]. By using a membrane-permeant Ca^2+^ chelator, TPEN (N,N,N′,N′-tetrakis(2-pyridylmethyl)ethylenediamine), we manipulated the luminal Ca^2+^, an approach taken previously in other systems for IP_3_Rs [13] and RyRs [24], and our data are consistent with a role for luminal Ca^2+^ in NAADP-regulated channel gating.

## 2 Methods

### 2.1 Homogenate preparation

Sea urchin egg homogenate was prepared as detailed [25]. Eggs from *Lytechinus pictus* were harvested by intracoelomic injection of 0.5 M KCl, collected in artificial sea water (ASW (mM): 435 NaCl; 40 MgCl_2_; 15 MgSO_4_; 11 CaCl_2_; 10 KCl; 2.5 NaHCO_3_; 20 Tris base, pH 8.0) and de-jellied by passage through 100-μm nylon mesh (Millipore). Eggs were then washed four times in Ca^2+^-free ASW (the first two washes containing 1 mM EGTA) and then washed in intracellular-like medium (GluIM (mM): 250 potassium gluconate; 250 N-methylglucamine (NMDG); 20 HEPES and 1 MgCl_2_, pH 7.2). Homogenization was effected with a glass Dounce tissue homogenizer in ice-cold GluIM supplemented with 2 mM MgATP; 20 U/ml creatine phosphokinase; 20 mM phosphocreatine; Complete™ EDTA-free Protease Inhibitor tablets (Roche). Homogenate (50%, v/v) was centrifuged at 13,000 × *g*, 4 °C for 10 s and the supernatant stored at −80 °C. On the day of use, an aliquot of homogenate was sequentially diluted in equal volumes of GluIM containing the ATP regenerating system over a period of 3 h at 17 °C to give a 2.5% (v/v) final concentration.

### 2.2 Fluorimetry

#### 2.2.1 Ca^2+^ release

All fluorimetry was conducted at 17 °C in a microcuvette containing a magnetic stir bar mounted in a Perkin Elmer LS-50B fluorimeter. Ca^2+^ release was measured in homogenate with 3 μM fluo-3 (excitation/emission: 506/526 nm) which was calibrated using the standard equation [Ca^2+^] = *K*_d_ × (*F* − *F*_min_)/(*F*_max_ − *F*), using a *K*_d_ of 0.4 μM; *F*_min_ and *F*_max_ were determined by addition of 0.5 mM EGTA and 10 mM Ca^2+^ respectively at the end of each run. TPEN (dissolved in ethanol) had no effect upon dye calibration (*F*_min_ and *F*_max_ values were 98 ± 5% and 101 ± 3% of ethanol controls respectively; *n* = 9, *P* > 0.5 paired *t* test). The upstroke kinetics were determined by linear regression of the raw fluorescence (in units (U)/s) normalized to the resting fluorescence (*F*_0_) to account for machine variability and therefore expressed as units•F_0_/s (U•F_0_/s).

#### 2.2.2 Acidic vesicle pH

We monitored luminal pH (pH_L_) as before [26]. 10 μM acridine orange was added to each cuvette immediately before each run and allowed to equilibrate (5–10 min) while the dye partitioned into acidic vesicles, as indicated by a gradual fall in fluorescence (acquisition wavelengths were the same as for fluo-3). An increase in fluorescence represents an increase in pH_L_. Data were expressed as a percentage of the maximum minus minimum fluorescence (the maximum was defined as the fluorescence after addition of 10 mM NH_4_Cl at the end of the run; this was equivalent to the pre-quench acridine orange fluorescence at the beginning of the recording [26]).

### 2.3 Data analysis and source of reagents

Representative traces are plotted as raw fluorescence (relative fluorescence units, RFU) against time. Data are expressed as the mean ± SEM. Two data sets were compared using Student's *t* test, whereas multiple groups were analysed using ANOVA and a Tukey–Kramer or Dunnett's post-test. Data were paired where appropriate and significance assumed at *P* < 0.05. Graphs were annotated using the following conventions: *P* < 0.05 (*), *P* < 0.01 (**), *P* < 0.001 (***). Curve fitting was conducted using Graphpad Prism.

NAADP was enzymatically synthesised [25] or purchased from Sigma–Aldrich (Poole, Dorset, UK). IP_3_ was from LC Laboratories (Woburn, MA, USA). Acridine orange and fluo-3 (K^+^ salt) were from Invitrogen (Paisley, UK). Nigericin, TPEN, phenanthroline and potassium oxalate were from Sigma–Aldrich whilst ionomycin (free acid) was from Calbiochem-Novabiochem (Merck Biosciences, Nottingham, UK). All other reagents were of analytical grade.

## 3 Results

### 3.1 NAADP-induced Ca^2+^ release

**Fig. 1:**
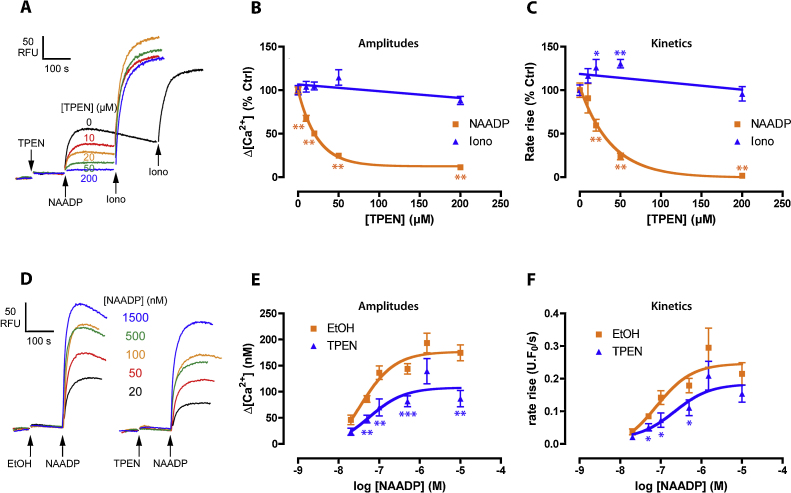
TPEN inhibits NAADP-induced Ca^2+^ release. (A) Different concentrations of TPEN (or 0.1% (v/v) ethanol vehicle) were preincubated for 2 min prior to addition of a sub-maximal concentration of NAADP (50 nM) followed by 0.5 μM ionomycin. Raw control Δ[Ca^2+^] was 79 ± 9 nM (NAADP) and 227 ± 18 nM (ionomycin). Summary of the effect of TPEN upon the amplitude (B) or kinetics (C) of NAADP- or ionomycin-induced Ca^2+^ release shown in (A). (D) Effect of a sub-maximal concentration of TPEN upon NAADP sensitivity. 0.1% (v/v) ethanol (EtOH) or 20 μM TPEN was preincubated for 2 min prior to the addition of different NAADP concentrations. Graphs summarizing the amplitudes (E) or kinetics (F) of the responses in (D). *N* = 9–16 (A–C), *n* = 5–9 (D–F). Significance was determined using a Dunnett's test versus 0 μM TPEN (B and C); Student's *t* test comparing EtOH and TPEN (E and F). NAADP data were fitted as a one-phase exponential decay (B and C) or a Sigmoidal concentration–response (E and F).

NAADP-induced Ca^2+^ release (NICR) in sea urchin egg homogenate was monitored fluorescently in the presence of ethanol vehicle or the membrane-permeant, low affinity (40–130 μM [27,28]) Ca^2+^ chelator, TPEN. TPEN crosses membranes rapidly to lower luminal free Ca^2+^ [28] and a 2-min preincubation with TPEN produced a substantial concentration-dependent inhibition of NICR, both in terms of amplitude and kinetics (IC_50_ of 17 and 27 μM respectively; Fig. 1A–C). This did not reflect a general perturbation by TPEN of Ca^2+^ release (or the assay) because the response to the Ca^2+^ ionophore, ionomycin, was essentially unaffected (Fig. 1A–C), as was the Ca^2+^ dye calibration (see Section 2). By varying the NAADP concentration (Fig. 1D–F), we found that the major effect of TPEN was to reduce the maximal extent of NICR without substantially altering the affinity of the receptors for NAADP (EC_50_ (95% confidence intervals) – amplitude: ethanol 32 nM (9–113 nM), TPEN 56 nM (7–47 nM); kinetics: ethanol 79 nM (17–370 nM), TPEN 218 nM (33–1460 nM)).

### 3.2 IP_3_-induced Ca^2+^ release

**Fig. 2:**
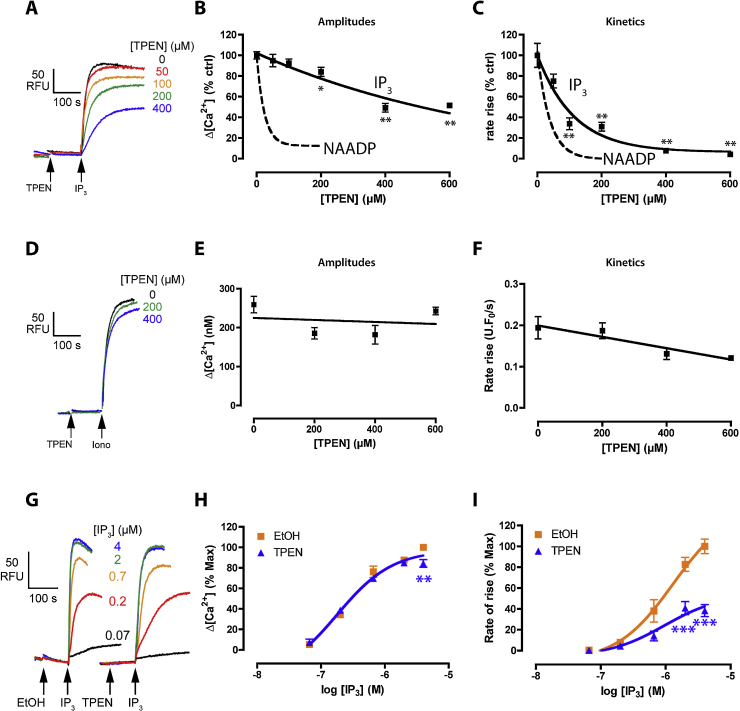
TPEN inhibits IP_3_-induced Ca^2+^ release. Different concentrations of TPEN (or 0.1% (v/v) ethanol vehicle) were preincubated for 2 min prior to addition of 4 μM IP_3_ (A–C) or 0.5 μM ionomycin (D–F). Raw control Δ[Ca^2+^] was 145 ± 6 nM (IP_3_) and 259 ± 21 nM (ionomycin). Summary of effect upon the amplitudes (B and E) or kinetics (C and F). The dashed lines (B and C) depict the NAADP curves in Fig. 1 for comparison. Significance was determined using a Dunnett test versus 0 μM TPEN. TPEN had no significant effect upon ionomycin responses (*P* > 0.05). IP_3_ data were fit as a one-phase exponential decay (B and C). Data are mean ± SEM of 3–8 experiments. (G–I) Effect of 200 μM TPEN (or 0.1% ethanol vehicle) upon the IP_3_ concentration–response. Data were normalized to the maximum response with 4 μM IP_3_ plus ethanol: raw control Δ[Ca^2+^] was 177 ± 18 nM (amplitude) and 0.578 ± 0.187 U F_0_/s (kinetics). IP_3_ data were fit with a Sigmoidal concentration–response (H and I) and ethanol and TPEN compared with a Student's *t* test (*n* = 5–12).

To ascertain whether the effect of TPEN was unique to NICR, we examined the effect of TPEN upon another channel regulated by luminal Ca^2+^, the IP_3_ receptor. Similar to NICR, IP_3_-induced Ca^2+^ release was also inhibited by TPEN but a major difference was that it required 3- to 30-fold higher concentrations of TPEN (estimated IC_50_ of 79 μM and 536 μM for kinetics and amplitudes respectively; Fig. 2A–C). Once again, these higher TPEN concentrations were essentially without effect upon ionomycin-induced Ca^2+^ release (Fig. 2D–F). As with NAADP, a sub-maximal concentration of TPEN predominantly affected the IP_3_ maximum and not the affinity, although the TPEN effect was more evident upon the kinetics than the amplitude (Fig. 2G–I): IP_3_ (EC_50_ (95% confidence intervals) – amplitude: ethanol 217 nM (46–1028 nM), TPEN 143 nM (50–413 nM); kinetics: ethanol 1303 nM (450–3774 nM), TPEN 953 nM (11–8106 nM)). The data are consistent with TPEN altering ER channel gating by chelating luminal Ca^2+^ as it does in other systems [13,24] and verify that the inhibition by TPEN is not peculiar to the NAADP-regulated channel.

### 3.3 TPEN and other ions

**Fig. 3:**
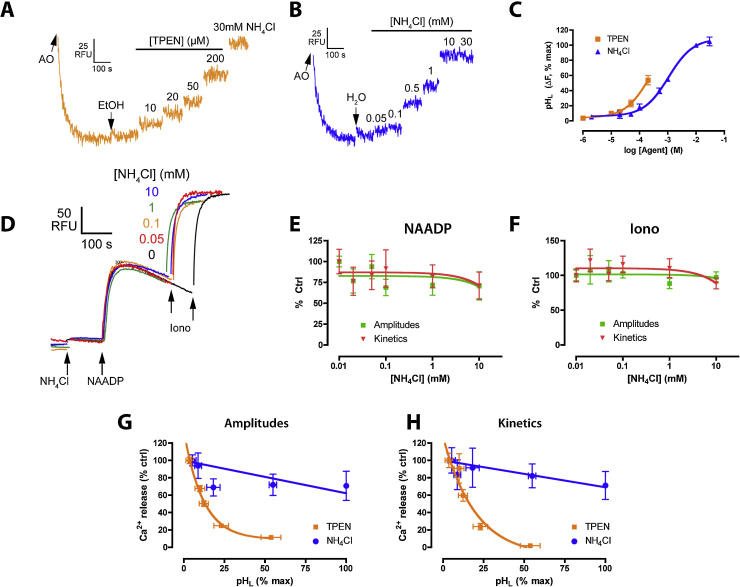
Comparison of the effects of TPEN and NH_4_Cl on luminal pH or NAADP-induced Ca^2+^ release. Luminal pH (pH_L_) was monitored using acridine orange (AO), panels A–C. Cumulative concentration–response curves to TPEN (A) or NH_4_Cl (B), and summarized in (C), where data were fitted as a Sigmoidal concentration–response. Effect of NH_4_Cl upon NAADP-induced Ca^2+^ release (D–F): different concentrations of NH_4_Cl were preincubated for 2 min prior to addition of sub-maximal NAADP (50 nM) and 0.5 μM ionomycin. Raw control Δ[Ca^2+^] was 96 ± 10 nM (NAADP) and 249 ± 22 nM (ionomycin). No significant effect of NH_4_Cl (*P* > 0.05) upon NAADP (E) and ionomycin (F) responses was observed (Dunnett's test). Data are mean ± SEM of 8–13 determinations. (G and H) For each concentration of TPEN or NH_4_Cl, the corresponding pH_L_ or Ca^2+^ signals were plotted to assess the relationship between the two parameters (including data from Fig. 1).

Whilst IP_3_ releases Ca^2+^ from the neutral ER, NAADP mobilizes Ca^2+^ from acidic Ca^2+^ stores which, in the sea urchin egg, appear to be the lysosome-related organelles, yolk platelets [29,30]. Since acidic Ca^2+^ store loading [11] and TPC channels [11,17,18,31] may be sensitive to luminal pH (pH_L_), we investigated whether the inhibition by TPEN was due to changes in pH_L_ rather than Ca^2+^. First, we monitored pH_L_ in NAADP-sensitive vesicles using acridine orange as reported previously [26]. TPEN did indeed raise pH_L_ slightly as judged by the increase in acridine orange fluorescence but with a lower potency than its effect upon NICR (estimated EC_50_ 222 μM; Fig. 3A and C). Although this 10-fold lower potency argued against pH_L_ as the factor underlying NICR inhibition, we directly tested whether an acute change in pH_L_ could modulate Ca^2+^ release by applying the base, NH_4_Cl. As expected, NH_4_Cl profoundly increased acridine orange fluorescence (pH_L_) with an EC_50_ of ∼1 mM (Fig. 3B and C) but, despite this, NH_4_Cl had no major effect upon NICR (Fig. 3D and E) or subsequent ionomycin-induced Ca^2+^ release from neutral stores (Fig. 3D and F). This suggested that TPEN does not act via increases in pH_L_ and this conclusion is reinforced by plotting the relationship between pH_L_ (acridine orange fluorescence) and Ca^2+^ release in the presence of TPEN or NH_4_Cl (Fig. 3G and H): although there is a tendency for higher pH_L_ to inhibit NICR (as seen with NH_4_Cl), it is clear that the effect of TPEN is greater than would be expected from an effect on pH_L_ alone. We conclude that TPEN does not act via pH_L_.

It is well documented that TPEN also binds to trace metal ions such as Zn^2+^ and Fe^2+^ with high affinity [27]; conceivably, TPEN could affect NICR by trace-metal chelation and as so a control for this, we tested the effect of phenanthroline which potently binds trace metals (*K*_d_s in the nanomolar to low micromolar range) but its *K*_d_ for Ca^2+^ (78–200 mM [32,33]) is 2000- to 5000-fold lower than that of TPEN. Table 1 shows that phenanthroline had no significant effect upon NICR amplitude or kinetics which contrasted with a TPEN positive control. We conclude that TPEN does not inhibit NICR by chelating trace metals.

### 3.4 TPEN and the Ca^2+^ electrochemical gradient

The rate and extent of Ca^2+^ release from any intracellular store is a function of the Ca^2+^ electrochemical gradient i.e. the Ca^2+^ concentration gradient plus the organellar membrane potential, Δ*ψ* [11]. Consequently, it was possible that TPEN acted by reducing this electrochemical gradient, most obviously by dramatically lowering the free luminal [Ca^2+^] in acidic Ca^2+^ stores. To test this, we reasoned that altering the Ca^2+^ electrochemical gradient would impact upon all Ca^2+^ release pathways, not just TPCs, and so we tested the effect of TPEN upon another pathway, the Ca^2+^ leak. Any Ca^2+^ leak can be unmasked by inhibiting the compensatory Ca^2+^ uptake; in acidic Ca^2+^ stores, the H^+^ gradient (ΔpH) facilitates Ca^2+^ uptake [11] and so we collapsed ΔpH as a means of indirectly inhibiting Ca^2+^ uptake. Note that sea urchin egg acidic vesicles are not very H^+^ “leaky” [26,29,30] so we could not use the V-H^+^-ATPase inhibitor, bafilomycin A1, to passively collapse ΔpH. Instead, we used nigericin (an electroneutral, K^+^/H^+^ exchange ionophore) to rapidly dissipate the ΔpH of acidic vesicles without substantially altering their Δ*ψ* [11]. In this way, Ca^2+^ release via the basal leak pathway can be revealed.

**Fig. 4:**
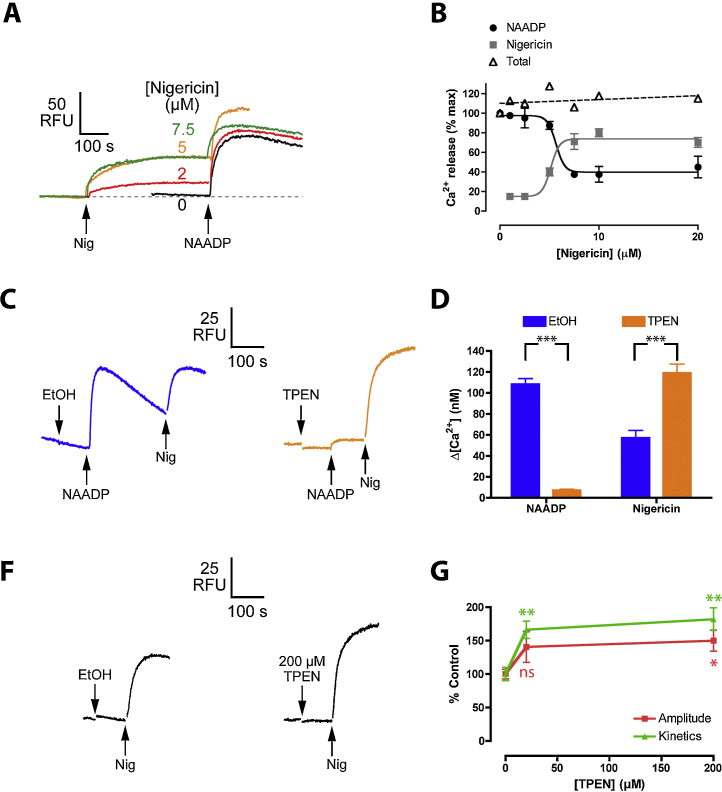
TPEN does not inhibit the acidic store Ca^2+^ leak pathway. (A) Nigericin evokes Ca^2+^ release from the NAADP-sensitive store. Increasing concentrations of nigericin evoke Ca^2+^ release that progressively depletes the store released by 250 nM NAADP as indicated by the reduced response. (B) Summary of peak data. ‘Total’ refers to the summation of the Ca^2+^ release evoked by NAADP plus that evoked by nigericin (*n* = 3). (C and D) Effect of 200 μM TPEN upon the Ca^2+^ responses to sequential addition of 50 nM NAADP and 20 μM nigericin (nigericin). *N* = 9. Ethanol vehicle (EtOH). (F) Effect of TPEN upon the Ca^2+^ leak unmasked by 20 μM nigericin. (G) Summary of the effect of different TPEN concentrations upon the amplitude or kinetics of the nigericin-induced Ca^2+^ responses (*n* = 5–6). ns, not significant.

We have previously shown that nigericin collapses ΔpH in sea urchin egg acidic vesicles [26] and so we tested whether nigericin mobilizes Ca^2+^ from the NAADP-sensitive acidic vesicles. Increasing concentrations of nigericin promptly released Ca^2+^ and the size of the NAADP-sensitive store was subsequently assessed by application of a high concentration of messenger: we observed a reciprocal relationship between the extent of nigericin- and NAADP-induced Ca^2+^ release (Fig. 4A and B) consistent with both agents acting on common Ca^2+^ stores. Conversely, mobilization of stores by NAADP reduced the response to nigericin (see below). The reduction of the response to NAADP was not simply a consequence of nigericin raising the baseline because thapsigargin, which also elevates Ca^2+^ by mobilizing the ER Ca^2+^ stores, has no effect upon NICR [8].

Having established that nigericin mobilized NAADP-sensitive stores, we turned to the effect of TPEN. In control experiments, consecutive responses to a sub-maximal concentration of NAADP and nigericin were measured in vehicle-treated homogenate; consistent with a common Ca^2+^ store, the response to nigericin was small when added after NAADP (Fig. 4C and D). In the presence of TPEN, NICR was almost completely inhibited but when nigericin was then applied, a substantial Ca^2+^ release was observed that was enhanced to approximately the same size as the control NAADP response (Fig. 4C and D). That is, even when the response to NAADP was blocked by TPEN, nigericin could still mobilize this Ca^2+^ store.

We then directly assessed the effect of TPEN upon nigericin-induced Ca^2+^ release (Fig. 4F and G). TPEN did not inhibit the nigericin responses, and, in fact, slightly enhanced the leak pathway, both in terms of its kinetics and amplitude (Fig. 4G). This indicates that the Ca^2+^ leak pathway unmasked by nigericin is manifestly different from that recruited by NAADP. Taken together, the data suggest that TPEN does not exert its effect by dramatically altering the Ca^2+^ electrochemical gradient and therefore we conclude that TPEN alters NAADP-regulated channel (TPC) gating.

## 4 Discussion

The idea that the degree of Ca^2+^ store filling (i.e. luminal [Ca^2+^]) modulates resident receptor channels in the store membrane has been with us for many years, applied first to RyRs and then later to IP_3_Rs [6,12]. Since then, other Ca^2+^ homeostatic proteins have emerged that tailor their activity to the luminal Ca^2+^ content such as SERCA [34] and STIM1 [35] and so the view that NAADP-regulated channels were, according to some criteria, insensitive to luminal Ca^2+^ singled them out as unique [9]. Unfortunately, this study was flawed because it was not then known that NAADP targets acidic Ca^2+^ stores and the luminal Ca^2+^ was manipulated with ionomycin [9] which does not act at acidic Ca^2+^ stores [36]. Given that there have been very few studies that have probed this issue, we have adopted a different strategy, using TPEN as a luminal Ca^2+^ chelator.

### 4.1 TPEN and acidic Ca^2+^ stores

To the best of our knowledge, TPEN has not been previously used to investigate NICR or indeed Ca^2+^ release from any acidic store. The sea urchin egg homogenate has proven an excellent system in which to investigate fundamental properties of NICR owing to its ease of use and access to the cell ‘cytosol’ [25]. TPEN exhibits a low affinity for Ca^2+^ (40–130 μM [27,28]) that precludes its buffering the cytosol (nM to μM) but favours buffering the higher Ca^2+^ range in internal stores (μM to mM) [11] and it has been used to manipulate the free [Ca^2+^] in the ER and given insights into RyR and IP_3_R gating [13,24] as well as store-operated Ca^2+^ entry [28,37].

First, does TPEN actually enter the acidic vesicle lumen? The fact that it promptly and persistently increases pH_L_ (acridine orange fluorescence) (Fig. 2) is consistent with such entry, and this pH_L_ change could either be a direct result of TPEN acting as a base or a secondary consequence of its chelating luminal Ca^2+^ (which disturbs the equilibrium between Ca^2+^ and H^+^ bound to the endogenous polyanionic matrix [26,30]). Moreover, we are confident that TPEN does not buffer extravesicular (“cytosolic”) Ca^2+^, not just because of its high *K*_d_ but also because (i) it does not alter basal [Ca^2+^]; (ii) it does not affect ionomycin- or nigericin-induced Ca^2+^ release; (iii) the Ca^2+^ calibration parameters *F*_min_ and *F*_max_ are unaffected. Therefore, the inhibition of NICR is not merely an artefact of “cytosolic” Ca^2+^ buffering.

Logically, it follows that TPEN must be interfering with the NAADP-regulated TPC itself or with the electrochemical driving force for Ca^2+^ release. Dealing with the latter, if the components of the electrochemical potential (free luminal [Ca^2+^] or Δ*ψ*) are substantially altered by TPEN, this would abrogate Ca^2+^ egress from the store. Our data suggest that this is not the case because Ca^2+^ release by ionophores would also be subject to the same thermodynamic constraints and yet the responses to ionomycin (neutral stores) and nigericin (acidic stores) were not reduced by TPEN.

### 4.2 Mechanism of action

We presented evidence that TPEN does not act by altering pH_L_ or heavy metals, but TPEN has also been reported to inhibit SERCA [38], as well as to activate [38,39] or inhibit [24] RyRs (depending on the TPEN concentration). However, these sites of action cannot underlie the TPEN effect on NICR because agents that selectively affect RyRs or SERCA do not alter NICR in egg homogenate [8,40], and besides, the affinity of SERCA for TPEN is ∼30-fold lower than towards NICR [39].

To offset concerns about TPEN pharmacology, we attempted to use a chemically dissimilar Ca^2+^-binding agent, oxalic acid, which also loads into the lumen of Ca^2+^ stores [41]. Like TPEN, oxalate inhibited NICR (data not shown). Unfortunately, oxalate also inhibited ionomycin-induced Ca^2+^ release and Ca^2+^-dye calibration (data not shown) and so we could not differentiate between potentially real luminal effects upon NICR and increased ‘cytosolic’ Ca^2+^ buffering by high concentrations of oxalate.

The fact that TPEN also altered IP_3_-induced Ca^2+^ release (albeit with a lower sensitivity) indicated that the effect is not peculiar to NAADP-regulated channels. Since TPEN does not directly block IP_3_Rs [42], it is consistent with an effect via luminal Ca^2+^ [13]. Nevertheless, the details of any effect of luminal Ca^2+^ upon IP_3_ receptor gating remain unclear (reviewed in [12]) and so our conclusion that luminal Ca^2+^ modulates IP_3_-induced Ca^2+^ release without altering IP_3_ sensitivity is not without precedent. We propose that this mechanism is shared by NAADP-gated channels.

Since TPEN did not grossly alter the Ca^2+^ electrochemical gradient in either neutral or acidic Ca^2+^ stores (as concluded from the ionomycin/nigericin experiments), there would have to be a steep relationship between luminal Ca^2+^ and channel (IP_3_R or TPC) opening, i.e. chelating Ca^2+^ over a narrow range alters channel function. Furthermore, modulation by luminal Ca^2+^ would have to be different between sea urchin and human because NAADP affinity was shifted by luminal Ca^2+^ in human TPC2 [17] whereas TPEN did not change the affinity in sea urchin. Clearly, only lipid bilayer studies will be able to unequivocally determine whether the sea urchin TPCs are regulated by luminal Ca^2+^ (as are human TPC1 [18] and human TPC2 [17]) and we cannot formally exclude the possibility that TPEN affects TPCs directly.

In summary, we show that the luminal Ca^2+^ chelator, TPEN, is a potent and effective inhibitor of NAADP-induced Ca^2+^ release. At the very least, this demands caution in interpreting effects of TPEN in biological systems, but it is also consistent with luminal Ca^2+^ being an important cofactor for NAADP-regulated TPCs. The regulation of IP_3_Rs and RyR by luminal Ca^2+^ priming sets a precedent for TPCs: perhaps TPC gating by NAADP could likewise be dynamically set by the luminal Ca^2+^ concentration which opens up the possibility of Ca^2+^ uptake from other sources (e.g. ER or Ca^2+^ influx) priming NAADP-sensitive stores.

## Figures and Tables

**Table 1:** Heavy metal chelation by phenanthroline does not affect NAADP-induced Ca^2+^ release.

Addition	Amplitude (Δ[Ca^2+^], nM)	Rate of rise (U/s)
0.1% ethanol	121 ± 2	0.187 ± 0.027
20 μM phenanthroline	126 ± 4	0.162 ± 0.015
200 μM phenanthroline	128 ± 3	0.163 ± 0.018
200 μM TPEN	10 ± 3**	nd

Amplitude of 50 nM NAADP-induced Ca^2+^ release 2 min after the addition of chelators or ethanol vehicle. Phenanthroline had no effect upon the basal [Ca^2+^] nor the response to NAADP. The small rise in the presence of TPEN was lost in the addition artefact and so the initial kinetics could not be determined (nd). Data are mean ± SEM of 6 determinations.

** *P* < 0.01, Dunnett's test versus ethanol.
